# Transmission of Tuberculosis by Vaccination

**Published:** 1881-12-20

**Authors:** 


### TRANSMISSION OF TUBERCULOSIS BY VACCINATION.

At a recent meeting of the Paris Academy of Sciences, M. Toussaint communicated some important results of his investigations into the microbial nature of tuberculosis. He had already succeeded, as Klebs and Cohnheim have, in cultivating these organisms, and had found that two drops of the liquid of the fourth artificial cultivation, inoculated into pigs, rendered them speedily and completely tuberculous. He then vaccinated a cow in an advanced stage of tuberculosis with lymph absolutely pure. The vesicles progressed normally, and with the lymph obtained from them he vaccinated different animals, all of whom subsequently became tuberculous. The significance of these experiments can scarcely be overrated, for though a judicious vaccinator would not use lymph taken from a child who exhibited already evidence of the disease, the chances of cows in whom spontaneous vaccinia may appear, and whose lymph would at the present time be eagerly sought after, being, like so many of their species, tuberculous, are great; and it would seem, in consequence, that the dangers of animal vaccination may be greater than those of human, which are supposed to be avoided by having recourse to the cow. As many who believe in the existence of a tuberculous microbion have failed in their attempt to cultivate it, we shall, at the earliest opportunity, give the details of M. Toussaint's methods.—*Medical and Surgical Reporter.*

